# Spondylosis in Horses: Clinical Features, Diagnostic Imaging Findings, Treatment and Outcome in 13 Horses

**DOI:** 10.1002/vms3.70196

**Published:** 2025-03-20

**Authors:** Claudia de Secondi, Federica Cantatore, Marco Marcatili, Marianna Biggi, Jonathan Withers, Donatella de Zani, Davide Zani

**Affiliations:** ^1^ Pool House Equine Hospital, IVC Evidensia Fradley UK; ^2^ Department of Veterinary Medicine and Animal Sciences University of Milan Lodi Italy

**Keywords:** back pain, horses, thoracic spondylosis, vertebral bodies

## Abstract

**Background:**

Back pain is a debilitating condition hampering horses’ athletic careers. Thoracic spondylosis (TS), a known cause of back pain, leads to osteophytes formation across intervertebral joints. In horses, TS is poorly reported, with anecdotal signs and response to treatment.

**Objectives:**

To report clinical presentation, diagnostic imaging findings, treatment and outcome in horses with TS.

**Methods:**

The clinical records of horses diagnosed with TS between 2010 and 2023 were reviewed. Signalment, clinical and imaging findings, treatment, and outcome were analysed. Thoracic spondylosis was graded from 1 to 5. Grades, concurrent pathologies, treatment, and outcome were assessed. The median TS grade and number of lesions and outcome were compared using the Mann–Whitney test.

**Results:**

Thirteen horses met inclusion criteria, eight of which performed a discipline involving jumping. All horses exhibited signs consistent with back pain. Thoracic spondylosis sites varied from 1 to 6 (mean 2) with a total of 32 lesions. The most affected site was T13–T14. Five horses had concurrent dorsal spinous processes impingement and three were lame. Treatment included physiotherapy, tiludronate, anti‐inflammatory and extracorporeal shockwave therapy. Long‐term follow‐up (>12 months) was available for 11 horses: the outcome was poor in seven horses, good in one and excellent in three. No statistically significant association was found between TS grade (*p* = 0.4), number of lesions (*p* = 0.2) and outcome categories.

**Conclusions:**

Although rare, TS can cause back pain. The outcome is generally poor, but some horses may continue athletic activity despite severe lesions.

## Graphical abstract

The aim of this study is to describe for the first time clinical signs, treatment and long‐term outcomes of horses diagnosed with thoracic spondylosis. Of the 13 horses that met the inclusion criteria, four were able to continue their athletic careers with good results, after treatment and rehabilitation.

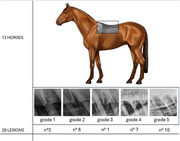

## 1 Introduction

Back pain is a common concern in sport horses, which can be career limiting (Xie, Colahan, and Ott 2005, Riccio et al. 2018). Reported causes for back pain include impingement of the dorsal spinous processes (DSPs; ‘kissing spine’), osteoarthritis of the synovial intervertebral joints, soft tissue injuries and spondylosis (Jeffcott 1979; Ranner, Gerhards, and Klee 2002; Walmsley et al. 2002).

Spondylosis is a degenerative condition causing ankylosis of the vertebral joints (Haussler, 1999). It is mostly present on both the left and right sides of the ventral midline on the thoracic vertebral bodies and the lateral surface of the lumbar vertebral bodies (Meehan et al. 2009; Scilimati et al. 2023). Spondylosis results in the development of osteophytes forming across the intervertebral joint, often affecting more than one consecutive joint (Meehan et al. 2009; Van Wessum 2020). In horses, spondylosis is commonly located between the 10th and 14th thoracic vertebrae (Meehan et al. 2009; Van Wessum 2020). However, this finding is likely linked to the limitations encountered in imaging the equine lumbar spine.

Despite its pathogenesis being still unclear, it has been hypothesised that the mechanical stress on the fibres joining the peripheral intervertebral disc and the vertebral endplate (Sharpey's fibres) may predispose to the condition. Torsional forces on the *annulus fibrosus* might stimulate osteoproduction, leading to the formation of osteophytes, which may reduce spinal mobility and, in the most advanced cases, may result in ankylosis (Meehan et al. 2009; Van Wessum 2020). The incidence in horses varies substantially depending on the methods of examination. It has been reported to affect around 3% of the horses with clinical signs of back pain investigated by radiography and scintigraphy (Jeffcott 1980; Meehan et al. 2009). However, when necropsy or computed tomography was used, spondylosis was diagnosed, respectively, in 36% and 42.5% of the cases. (Scilimati et al. 2023; Spoormakers et al. 2024; Townsend et al. 1986).

In people, the prevalence in patients over 50 years of age can reach up to 80%, with a higher incidence in men performing heavy physical activity. Despite the pathogenesis not having been clarified yet, abnormalities in the peripheral fibres of the *annulus fibrosus* could potentially lead to anterolateral disc displacement, traction on its osseous attachment and consequent osteophyte development, which occurs several millimetres away from the disco‐vertebral junction (Resnick 1985). In dogs, the term ‘*spondylosis deformans*’ is preferred, and the incidence varies between 17.8% and 75%. Correlation with Hansen type II *nuc* *leus polposus* herniation has also been hypothesised (Levine et al. 2006). A hereditary component is likely in dogs based on breed predisposition (Ihrke et al. 2019; Levine et al. 2006; Morgan, Ljunggren, and Rosemary 1967).

The clinical significance of spondylosis in horses remains uncertain (Meehan et al. 2009), with most studies reporting horses with either absent or mild signs of back pain, such as reduced back mobility (Haussler 1999, Denoix 2011). Other authors hypothesise severe clinical signs, including ataxia, following nerve root compression (Eskonen, Ruohoniemi, and Karkamo 2007). There are currently no specific treatments for horses. However, rest, systemic non‐steroidal anti‐inflammatory drugs and tiludronate are often used (Driver et al. 2018).

In horses, spondylosis remains a poorly reported condition and its presenting signs and response to treatment remain anecdotal and based on clinical experience. Therefore, the aim of this case series is to document clinical and diagnostic imaging findings, therapy and long‐term outcomes of horses radiographically diagnosed with thoracic spondylosis (TS) as part of poor performance investigations.

## 2 Methods

The clinical records of two Equine Hospitals (Pool House Equine Hospital and Department of Veterinary Medicine and Animal Sciences of the University of Milan–blinded for review) were retrospectively reviewed for adult horses presented for poor performance, behavioural issues, or back pain and subsequently diagnosed with TS between September 2010 and August 2023 following radiographic evaluation of the back. Horses' signalment, presenting complaint, clinical findings, imaging findings, treatment and outcome were recorded.

Cases were included if radiographs of the back, including thoracic vertebral bodies (laterolateral projections) and DSPs (laterolateral projections) from T8 to T17, were available and of diagnostic quality. When available, radiographs of lumbar vertebral bodies and DSPs (laterolateral), as well as oblique views of thoracic and lumbar articular process joints (latero20° ventral‐laterodorsal oblique views) and ultrasonographic images of the back and pelvis (including ultrasonographic evaluation of the ventral aspect of the fifth and sixth lumbar vertebrae, the first sacral vertebra and their intervertebral discs, the sacroiliac joints, the intertransverse joints and the *pubis* performed *per rectum*), were evaluated and reviewed. Information was retrieved from the management systems in use at the two sites (Assisi Veterinary System, Practice Point, Crewe, Cheshire, UK and Provet Cloud, Nordhealth Ltd., Helsinki, Finland) by one of the authors (CDS). Data regarding horses' signalment (breed, age and sex), their use, the presenting complaint, the results of the orthopaedic evaluation, such as the presence of lameness, lame limb/ limbs and lameness grade, the results of diagnostic analgesia, TS sites, TS grade, concurrent orthopaedic pathologies, treatment and outcome were inserted into an electronic spreadsheet (Microsoft Excel 16.54) and the cases were identified using consecutive numbers to anonymise them.

Horses underwent a complete clinical examination on admission at the hospital. Physical and dynamic examination was performed by a veterinary surgeon experienced in equine orthopaedics, using a systematic approach as described in the literature (Denoix and Dyson 2011). The horses were first visually inspected; then the thoracolumbar region was palpated to detect painful reactions, and it was mobilised to assess range of movement. The horse was then observed moving at a walk and trot in a straight line and on the lunge, both on hard and on soft surfaces. Subsequently, the horse was cantered on the lunge on soft ground on both reins. When lameness was detected, it was graded on a scale ranging from 0 to 5 according to the American Association of Equine Practitioners (AAEP). Diagnostic analgesia was performed when considered appropriate by the clinician.

All radiographic images were acquired with the following equipment: Ultrapower 400, veterinary x‐rays, Princes Risborough, Buckinghamshire, England and Rotanode 150 kV, Toshiba, Shimoishigami, Otawara‐shi, Japan.

Scintigraphic examination was performed in one case using a customised Spect Prism 2000XP (Picker International, Highland Heights, Ohio) over 90 s, using a high‐resolution collimator, 150 min after intravenous injection of 1 GBq/100 kg of 99m‐technetium‐methylene diphosphonate.

All the images available for the cases, including radiographs of the thoracolumbar region and scintigraphy scans of the back, were retrospectively reviewed by a blinded operator, ECVDI board diplomate (MB). First, TS was graded using a previously described radiological grading system comprised of grades from 0 to 5 (Meehan et al. 2009):

- 0: no osteophytes present
- 1: osteophytes from one vertebral body, not bridging intervertebral space
- 2: osteophytes from two adjacent vertebral bodies, not meeting
- 3: osteophytes from two adjacent vertebral bodies, meeting but without evidence of increased opacity at opposing borders
- 4: osteophytes from two adjacent vertebral bodies, meeting and with evidence of increased opacity at opposing borders
- 5: complete bridging of the intervertebral space

Other radiographic changes within the thoracolumbar spine, such as DSP pathology, were also recorded. Impingement of the DSPs pathology was graded according to a 0–7 scale previously reported in the literature (Zimmerman, Dyson, and Murray 2012). Radiographic changes at the level of the thoracolumbar articular process joints, such as sclerosis, osteolysis, periarticular bone modelling, dorsal extension of the joint margin and narrowing or complete loss of the articular space consistent with osteoarthritis, were also noted (Denoix, 2011). If a fracture was observed, this was described, including the affected area, extension and radiographic appearance.

Treatment was recorded. If the horse was referred back to the primary treating veterinary surgeon, they were contacted telephonically to obtain the required information.

Information regarding the long‐term outcome after the diagnosis (>12 months) was retrieved from the practice management system or by telephone questionnaire with the owner (Supplement 1). Outcome was evaluated as ‘excellent’ if the horse returned to the same or higher level of activity, ‘good to fair’ if the horse returned to some activity but at a lower level, ‘poor’ if the horse was retired because it could no longer sustain athletic activity as a result of back pain.

### 2.1 Statistical Analysis

After each TS site was graded, the grades were added and the sum of the grades of each horse was considered its total radiological grade. The outcomes were then divided into two categories (‘positive’, including excellent and good to fair outcomes, and ‘negative’ for poor outcomes) and for both categories, the mean value of the radiologic grade and number of lesions was obtained. Afterwards, the TS radiologic grade and lesion number were compared to the outcome, and the statistical significance of such results was assessed using a non‐parametric test such as the Mann–Whitney test using R (v4.2.1, R Core Team (2023). R: A language and environment for statistical computing. R Foundation for Statistical Computing, Vienna, Austria. https://www.R‐project.org/). A *p* value of <0.05 was considered statistically significant.

## 3 Results

### 3.1 Cases

Of the 1348 horses presented for poor performance, behavioural issues or back pain in the two hospitals, 13 horses met the inclusion criteria, which represent less than 1% of the cases. The mean age was 11.8 years old (SD 2.6 years), the age range being 7–17 years. Eleven horses were over 9 years of age. Eight of these horses were mares, the remaining five horses were geldings. Four horses were Thoroughbreds or Thoroughbred crosses, four were Warmbloods and two were Irish Sport Horses. Eight horses performed a discipline involving jumping: four were show jumpers, four were eventers and one horse was a hunter. The remaining horses were used either in dressage (1) or general riding (4). One horse performed multiple disciplines (case 7).

### 3.2 Presenting Signs

Two horses were referred exclusively for imaging of the back and no clinical information was available.

All horses showed clinical signs associated with back pain, the most common of which were: three horses were painful on palpation of the back, two showed poor epaxial muscle development, four were reluctant to go forward when ridden, three were reluctant to canter and four were showing signs when ridden, such as tail wishing, rearing and bucking.

Four horses were lame on admission and underwent diagnostic analgesia. Three of them were positive for distal limb diagnostic analgesia. These data are available in Supplement 1.

### 3.3 Imaging Findings

**FIGURE 1:**
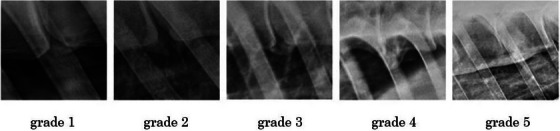
Image illustrating spondylosis grades according to Meehan et al. (2009).

In all cases, laterolateral radiographic imaging of the vertebral bodies from T8 to T17 was available. Imaging of the lumbar vertebral bodies was not available in any of the cases. The number of sites of spondylosis varied from 1 to 6 (mean 2). The majority of the horses (eight) had a single site of spondylosis. Of these, one horse had a grade 1 lesion, three horses had a grade 2 lesion, one horse had a grade 3 lesion and three had a grade 4 lesion. In all horses with more than one lesion, they were on adjacent vertebral bodies. A total of 29 lesions was identified and graded: three (10.3%) were grade 1, eight (27.6%) were grade 2, one (3.4%) was grade 3, seven (24.1%) were grade 4 and 10 (34.5%) were grade 5. Lesion grading and distribution are summarised in Figure 1.

**FIGURE 2:**
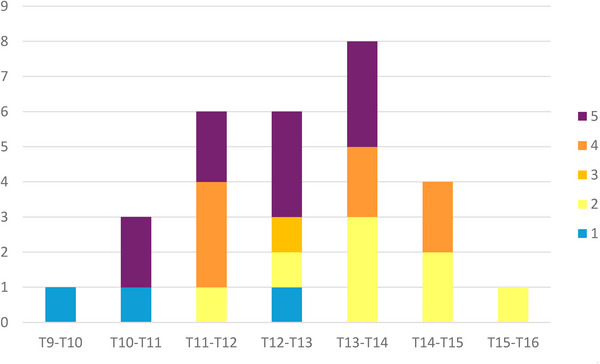
Location and grading of spondylosis lesions. On the Y axis the number of lesions is reported, on the X axis there is the location of the lesions (intervertebral joints from T9 to T17). Different colours represent different lesion grades.

**FIGURE 3:**
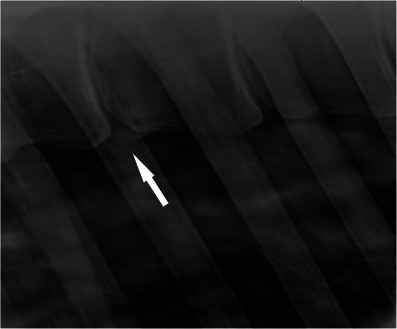
Laterolateral radiographic view of the vertebral bodies of a 12‐year‐old Thoroughbred cross mare (case 3). Cranial is to the left of the radiograph. There is one grade 1 spondylosis lesion (white arrow).

**FIGURE 4:**
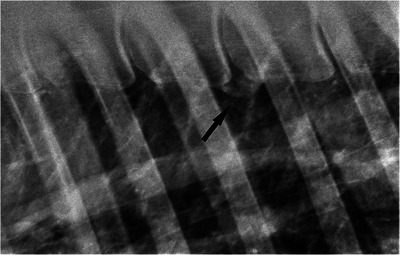
Laterolateral radiographic view of the vertebral bodies of a 14‐year‐old Thoroughbred cross mare (case 9). Cranial is to the left of the radiograph. There is one grade 3 spondylosis lesion (black arrow).

The most commonly affected site was between the 13th and 14th thoracic *vertebra* (T13–T14, eight lesions over 29 lesions in total), while six lesions occurred between the 12th and 13th thoracic *vertebra* (T12–T13) and four lesions between the 14th and 15th thoracic *vertebra* (T14–T15). The majority of grade 5 lesions occurred between T11 and T14 (eight lesions out of 10 in total) and eight of the 20 lesions observed between T11 and T14 were grade 5 (Figures 2, 3, 4).

For 11 out of 13 horses, radiographs of the DSPs were available. Nine horses (cases 1, 2, 4, 5, 6, 7, 8, 10 and 11) had at least one site of grade 1 DSPs pathology (mean number of sites was 4.4, ranging between 1 and 9). Mean total radiological grade per horse was 8.7, ranging between 1 and 21. Two horses (5 and 11) underwent diagnostic analgesia at this level. Local anaesthetic (60 mL of mepivacaine) was injected parasagittal to the lesions as described in the literature (Denoix, 2011). Horses were re‐evaluated on the ground and during ridden exercise 15 min after injection without significant improvement of the clinical signs and therefore this condition was considered not clinically significant at that stage. Grading and location of sites are summarised in Table 1. One horse (case 8) had one thoracic site of osteoarthritis of the articular facet joints (APJ) and smooth osseous remodelling on the dorsal aspect of DSPs (T15–T18) suggestive of enthesopathy of the supraspinous ligament. Ultrasonographic evaluation of the back revealed no ultrasonographic changes within the supraspinous ligament and allowed us to grade the right APJ between T15 and T16 as ‘mild’ according to Morgan et al. (2024). One horse (case 4) presented loss of echogenicity of the lumbosacral disc consistent with disc degeneration associated with small periarticular osteophytes of the sacroiliac joints consistent with bilateral sacroiliac joint osteoarthritis (grade 1 according to Tallaj et al. 2019), which were diagnosed by ultrasonographic examination *per rectum*. Three horses had orthopaedic pathologies affecting one or more limbs: one (case 1) had left forelimb enthesopathy of the origin of the accessory ligament of the deep digital flexor tendon and poor foot balance, one horse (case 8) presented bilateral dystrophic mineralisation of hindlimbs proximal suspensory ligaments, and one horse (case 12) suffered from bilateral forelimb suspensory ligament desmopathy.

**TABLE 1:** Summary of case data including breed, age, sex, presenting complaint, location and grade of TS lesions, treatment and outcome.

Case	Breed	Age	Sex	Use	Presenting complaint	Lameness (AAEP) and diagnostic analgesia	TS sites	Grade of spondylosis lesion on admission	Concurrent orthopaedic pathologies	Treatment	Outcome
**1**	ISH	11	M	General riding	Horse disunited in canter	NA	T11‐T12	4	LF enthesopathy ALDDFT + foot imbalance Four sites of DSPs pathology (T13–T17, one grade 1, three grade 2)	4 months rest, 4 months groundwork, physiotherapy	Excellent
**2**	Welsh Cob x show hunter	14	M	Dressage	Reluctant to canter on right rein	NA	T13–T16	2, 4, 4	Five sites of DSPs pathology (T14–T18, two grade 1, three grade 2)	Tiludronate	Excellent
**3**	TB X	12	M	Eventing	Recently not moving well	NA	T12–T13	1	NA	NSAIDs, 4 months hand walking, mesotherapy	Good
**4**	ISH	7	G	Eventing	Unwilling to go forward and stopping while hacking	RH 1/5, no diagnostic analgesia	T13–T14	2	Mild osteophyte right SI, loss of homogeneity of lumbosacral disc, one site of DSPs pathology (T16–T17, one grade 1)	Tiludronate, NSAIDs (phenylbutazone, 1 gr/day for 1 month), 2 months hand walking, physiotherapy	Poor
**5**	TB	11	G	General riding	Reluctance to canter, holding head up once ridden	NA	T13–T14	2	Five sites of DSPs pathology (T13–T18, one grade 1, three grade 2, one grade 3)	4 months rest, NSAIDs (phenylbutazone 2 gr/day, corticosteroid injection in 4 facet joints, mesotherapy	Poor
**6**	WB	13	G	Show jumping	Bucking when ridden, girthy	NA	T13–T14	4	Two sites of DSPs pathology (T17–L1, one grade 2 and one grade 3).	Tiludronate, 3 months rest, then ridden flatwork physiotherapy (water treadmill)	Poor
**7**	Dutch WB	10	M	Eventing, hunting	NA	NA	T11–T15	2, 4, 5, 5	One site of DSPs pathology (T16–T17, grade 1)	NA	NA
**8**	Sport Horse	12	M	Show jumping	Reluctance to jump, toe dragging	LH 2/5, positive to deep branch of lateral plantar nerve block	T13–T16	2, 2, 4	Eight sites of DSPs pathology (T11–L2, two grade 1, four grade 2, one grade 3 and two grade 4 sites) enthesopathy supraspinous ligament, hindlimbs proximal suspensory ligament desmitis. Osteoarthritis of T15 and T16 left articular facet	Tiludronate, extracorporeal shockwave therapy, hand walking	Poor
**9**	TB X	14	M	General riding	Rearing when tacked up and ridden	RF 3/5, positive to lateral palmar nerve block	T12–T13	3	DIPJ osteoarthritis	1 × tiludronate, NSAIDs (suxibuzone, 1.5 gr/ day for 2 weeks), physiotherapy	Poor
**10**	Unknown	17	M	General riding	NA	LF 2/5, positive to abaxial sesamoid nerve block	T11–T12	2	Five sites of DSPs pathology (T13–T18, five grade 2 sites) articular facet joints osteoarthritis C3–C7	1 × tiludronate, NSAIDs (phenylbutazone 1 gr/ day for 2 weeks), physiotherapy	Poor
**11**	TB	8	G	Eventing	Back pain	NA	T11–T12	4	Eight sites of DSPs pathology (T13–L2+L3–L4, two grade 1, two grade 2, two grade 3 and two grade 4 sites)	NSAIDs (phenylbutazone 1 gr/day for 4 weeks), physiotherapy	Poor
**12**	WB	12	G	Show jumping	Disunited in canter before jumps	NA	T9–T14	1, 5, 5, 5, 5	Forelimbs suspensory ligament desmopathy	Tiludronate, mesotherapy	Excellent
**13**	WB	12	M	Show jumping	NA	NA	T10–T16	1, 2, 5, 5, 5, 5	NA	NA	NA

Abbreviations: G, gelding; ISH, Irish Sport Horse; M, mare; NA, not applicable; TB, Thoroughbred; TBX, Thoroughbred cross; WB, Warmblood.

**FIGURE 5:**
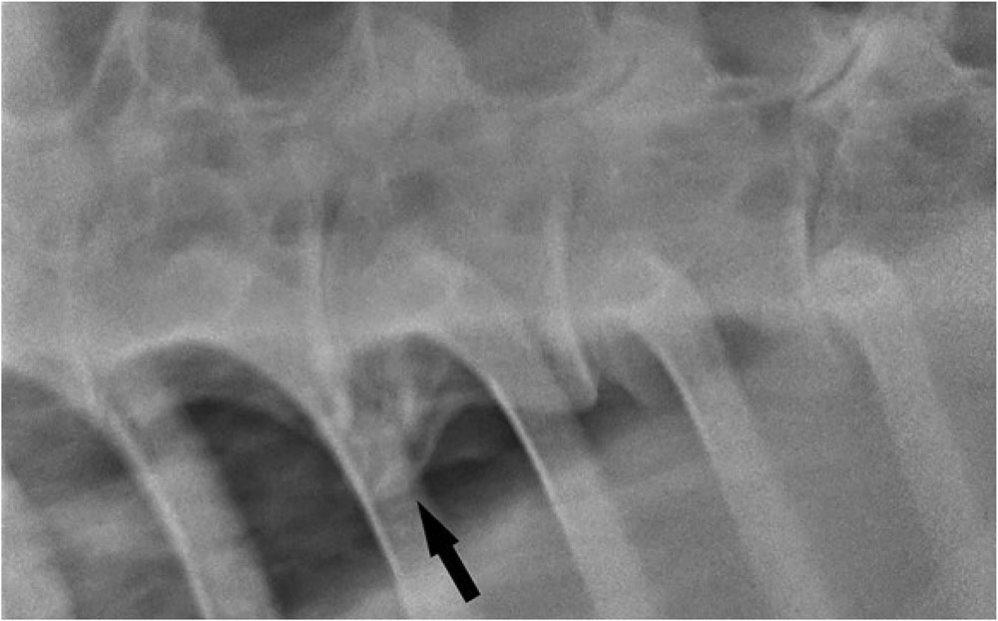
Laterolateral radiographic view of the vertebral bodies of a 7‐year‐old Irish Sport Horse gelding (case 4). Cranial is to the left of the radiograph. There is one grade 4 spondylosis lesion (black arrow).

Scintigraphic imaging of one horse (case 12) revealed mild IRU at the ventral aspect of the vertebral bodies between the ninth thoracic *vertebra* (T9) and T14 (more evident between T9 and T11). These findings were consistent with the radiographic imaging findings of grade 2 spondylosis lesion at T9–T10 and four grade 5 spondylosis lesions between T10 and T14 (Figure 5).

Follow‐up radiographic images were available for six horses and were obtained between 3 and 16 months following the initial examination. In three out of six horses, the lesions progressed. Out of eight total lesions observed in these radiographs, three progressed: one grade 4 lesion progressed to grade 5 in 3 months (case 2); a grade 1 lesion became grade 4 over 9 months (case 3); a grade 2 lesion progressed to grade 3 in 3 months (case 5). The remaining lesions were unaltered over a period between 3 and 16 months: two were grade 2 and three were grade 4.

### 3.4 Treatment

Treatment details were available for 11 horses out of 13 and are summarised in Table 1.

Seven horses received intravenous tiludronate (Tildren, Audevard, Clichy, France, 1 mg/kg), as a single infusion. Four horses received one infusion and three received a second infusion after 2 to 3 months. Six horses received non‐steroidal anti‐inflammatory drugs: five received phenylbutazone (Equipalazone, Dechra Pharmaceuticals PLC, Northwich, Cheshire, UK), one received suxibuzone (Danilon, Ecuphar NV, Oostkamp, Belgium) for 1 month. In three cases, mesotherapy was performed with various drugs, including a suspension of powdered *Sarracenia purpurin* (Sarapin, High Chemical Company, Levittown, PA), dexamethasone (Rapidexon, Dechra Pharmaceuticals PLC, Northwich, Cheshire, UK) and homeopathic preparations. Six horses were subjected to physiotherapy, and one received three sessions of extracorporeal shockwave therapy over the thoracolumbar area (Swiss DolorClast, ElectroMedical Systems SA, Nyon, Switzerland; 2000 pulses).

### 3.5 Outcome

Seven out of the 11 horses whose follow‐up was available had their outcome classified as ‘poor’, one as ‘good’ and three as ‘excellent’. Of the horses whose outcome was classified as ‘excellent’, one (case 1) was used for general riding, one (case 2) was an eventer and one (case 12) was a show jumper. The horse (case 3) whose outcome was classified as ‘good’ was an eventer, while of those whose outcome was ‘poor’, two were eventers (cases 4 and 11), three (cases 5, 9 and 10) were used in general riding and two (cases 6 and 8) were show jumpers. Horses whose outcome was categorised as ‘negative’ had a TS grade mean value of 3.6 and those whose outcome was ‘positive’ had a mean value of 9. Horses whose outcome was categorised as ‘positive’ had a mean number of lesions of 2.8, while for horses whose outcome was ‘negative’ it was 1.3. The possible effect of TS grade and number of lesions on outcome was formally evaluated and there was no statistically significant difference in terms of grade (*p =* 0.4) and number (*p =* 0.2) of TS lesions between outcome categories (‘positive’ vs. ‘negative’).

Outcome is summarised in Table 1.

## 4 Discussion

The purpose of this study was to document clinical and diagnostic imaging findings, treatment and outcome in a group of horses diagnosed with TS.

The horses included in this case series were adults and they were mainly used for jumping as previously described in the literature (Van Wessum 2020). Males and females were equally affected by spondylosis in some previous studies (Townsend et al. 1986), while some authors observed a higher prevalence in mares (Meehan et al. 2009). In the present study, mares were 61.5% of the population. This might be due to the small size of our population, or it might reflect a higher susceptibility in mares. Further studies involving a larger number of horses are necessary.

The clinical signs were unspecific but consistent with signs that have been referred to back pain (Riccio et al. 2018). The horses mainly showed discomfort when tacked up. During ridden exercise, some horses were bucking (2) and reluctant to canter and jump (3). The two horses that were exclusively diagnosed with spondylosis presented milder signs of back pain; however, it seems reasonable that the presence of multiple sources of pain would result in more severe discomfort. To accurately judge the clinical relevance of spondylosis, it would have been ideal to include horses affected by TS and without concurrent pathologies. However, this would have limited the number of horses and would not have reflected clinical practice.

In agreement with Townsend et al. (1986), lesions were predominantly found between T10 and T17 (28). In accordance with the recent literature, most lesions were found at the level of T13–T14 (8). Spondylosis was localised at T14 in 41.4% of lesions and T13 in 48.3%. The spine tract between T12 and T14 has the most rotational and lateral thoracolumbar mobility (Jeffcott, Kidd, and Bainbridge 2018; Townsend et al. 1983); therefore, the continuous strain on the peripheral intervertebral disc and ventral longitudinal ligament fibres may be the cause of this condition at this level.

In horses with multiple lesions, the grade and distribution of the lesions presented a similar trend to previous reports (Meehan et al. 2009): usually, the central site had a higher lesion grade, while the adjacent and probably newly formed ones presented lower grades. Out of the six horses with more than one lesion, five had higher‐grade lesions (grades 4 or 5). This may reflect the progressive nature of the disease and supports the hypothesised pathogenesis: the loss of range of movement (especially when higher‐grade lesions were found) in one motion segment alters the lateral and rotational forces applied to adjacent segments, consequently causing additional lesions, similarly to what happens in humans and to what was hypothesised in dogs (Levine et al. 2006). Eight cases had only one site of spondylosis and three of those had a grade 4 lesion. Although the limited number of cases does not allow us to draw definitive conclusions, we considered this finding unusual. In fact, we would have expected a high‐grade site of spondylosis would have impaired back functionality significantly, predisposing to the formation of spondylosis lesions in adjacent sites. It is however possible for such lesions to have been present but not detected, due to the absence, in some cases, of radiographic oblique views of the back. Nevertheless, a large portion of the back (thoracic spine cranial to T8 and caudal to T17, lumbar spine) could not be evaluated in the study due to the limitations present when performing radiographs of these areas in adult horses.

Despite lumbar spondylosis having been described in 7.7% to 10.3% of cases with and without signs of back pain (Meehan et al. 2009; Townsend et al. 1986), we have not observed any in this study. Most likely, this is due to the practical difficulties in radiographic assessment of the lumbar spine in the horse. This could also be due to spondylosis lesions developing on the lateral aspect of the lumbar vertebrae more than on the ventral aspect (Meehan et al. 2009; Scilimati et al. 2023). Laterolateral radiographic views of the back may limit the diagnosis of lateral spondylosis due to superimposition with the vertebral bodies.

Nine horses also presented one or more sites of impingement of DSPs, and one thoracic facet joint osteoarthritis. Most of the horses presented a low grade of impingement of the DSP. It has been reported that the lesions are clinically significant if their radiographic score is ≥3, and therefore it is likely to be an incidental finding (Zimmerman, Dyson, and Murray 2012). Although statistical analysis was not performed due to the limited number of cases, it appears that DPSs grade negatively impacted the outcome. In one horse (case 8), both the osteoarthritis lesions and one of the sites of DSPs pathology were in the same intervertebral joint as a grade 4 spondylosis lesion. It has been suggested that chronic cases of spondylosis may lead to facet joint cartilage damage, therefore causing DSPs to get closer (Van Wessum 2020), which may be the cause for the distribution of lesions in this case.

In the six cases in which follow‐up radiographic assessment was available, development of lesions was observed only in lower‐grade lesions (grade 3 or lower). One horse (case 4) presented abnormalities of the lumbosacral disc. Association of spondylosis with intervertebral disc degeneration has not been reported in this species, although this association has been made in humans (Seichi 2014) and dogs (Kranenburg et al. 2011; Levine et al. 2006; Morgan, Ljunggren, and Rosemary 1967) and further reports would be interesting to evaluate this aspect.

**FIGURE 6:**
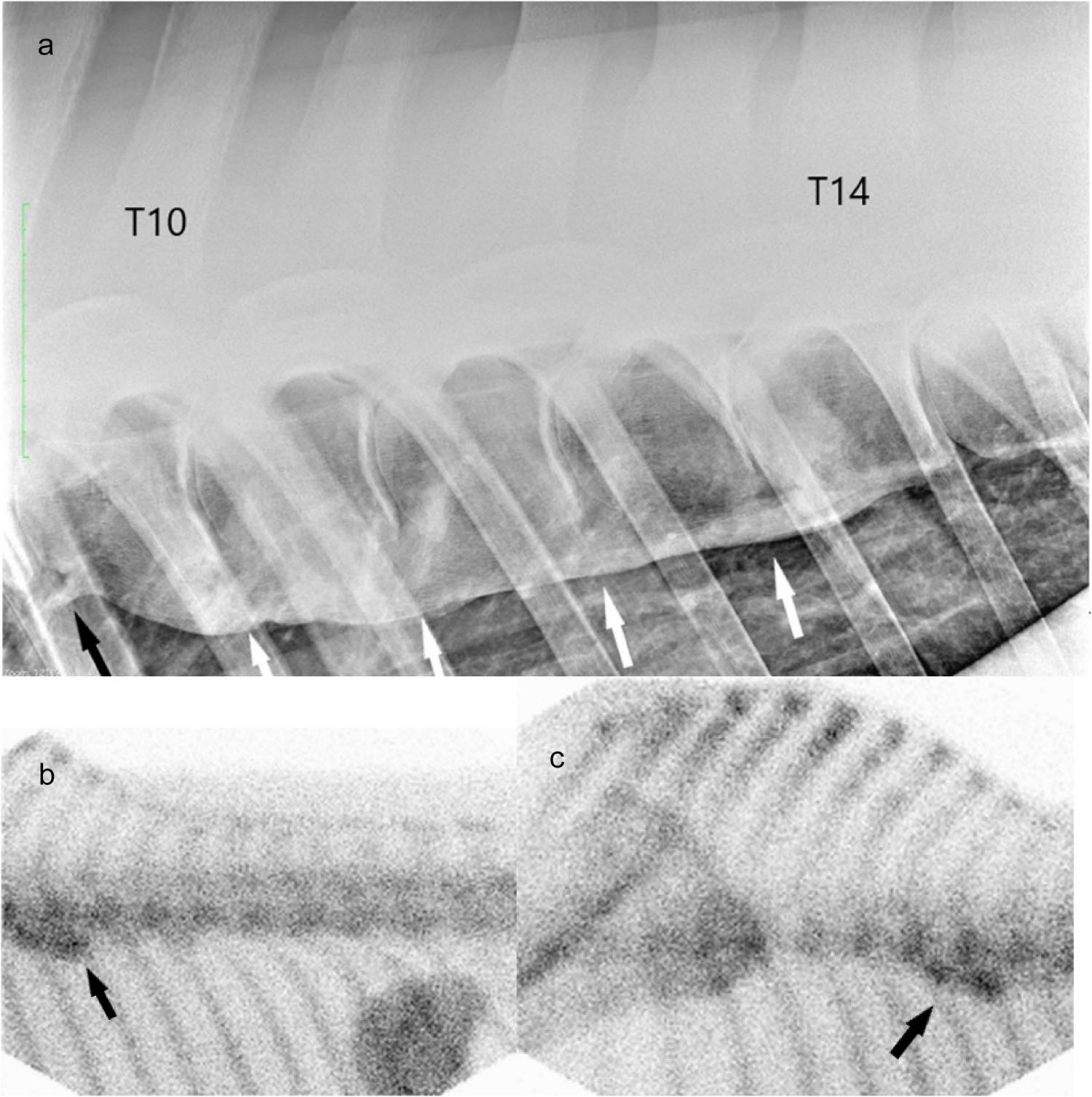
(a): Laterolateral radiographic view of the T9–T15 vertebral bodies of a 12‐year‐old Holsteiner gelding (case 12). Cranial is to the left of the radiograph. There are four grade 5 spondylosis lesions between T10 and T14 (white arrows), and a grade 2 lesion between T9 and T10 (black arrow), (b, c): Lateral bone phase scintigraph images of the thoracolumbar spine of the same horse showing IRU at the T9–T14 vertebral bodies (black arrows).

**FIGURE 7:**
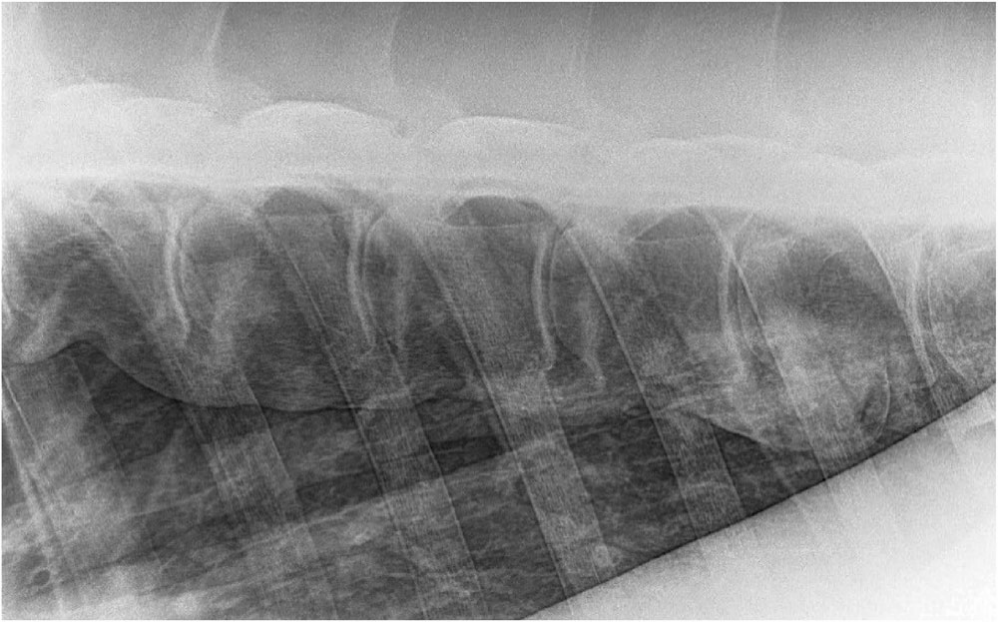
Laterolateral radiographic view of the T11–T17 vertebral bodies of a 12‐year‐old Holsteiner mare (case 13). Cranial is to the left of the radiograph. There are three grade 5 spondylosis lesions between T12 and T15, and a grade 4 lesion between T15 and T16, and a grade 2 lesion between T11 and T12.

**FIGURE 8:**
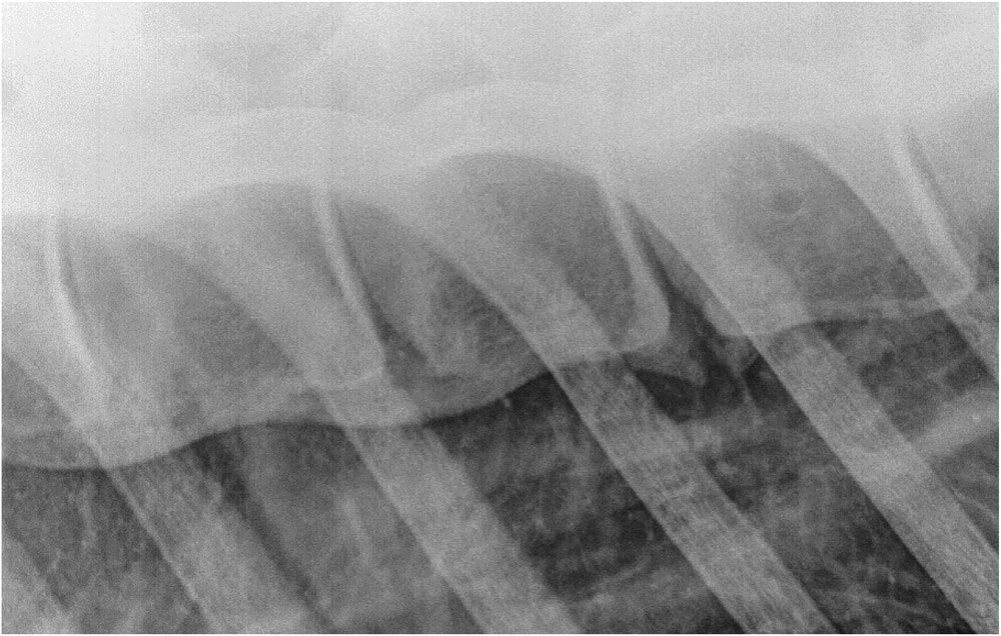
Right lateral radiographic view of the T12–T16 vertebral bodies of a 10‐year‐old Dutch Warmblood mare. Cranial is to the left. There are two grade 5 spondylosis lesions between T12 and T14, and a grade 2 lesion between T14 and T15.

The radiographic appearance of *spondylosis* grade 5 (Figures 6 and 7) is very similar to diffuse idiopathic skeletal hyperostosis (DISH). DISH is a systemic disease of the axial and appendicular skeletons leading to complete ossification of soft tissues such as the ventral longitudinal ligament (Marković et al. 2019). In dogs, the two diseases are differentiated only by *post‐mortem* macroscopic and histologic examination (Kranenburg et al. 2011). Due to this, we cannot exclude that some cases may have been misdiagnosed; however, DISH has been scarcely described in the horse (Marković et al. 2019) and therefore this is unlikely (Figure 8).

Scintigraphic imaging findings reflected what has been previously reported: spondylosis lesion grade is not proportional to IRU (Meehan et al. 2009). In both the present study and pre‐existing literature, scintigraphic imaging showed the greatest activity where lower‐grade lesions were located, although some was present at all sites. This result suggests more recent lesions may be developing faster, compared to the advanced ones. It is however a limitation of this study that scintigraphic imaging was only employed in one case (case 12).

Unfortunately, there was no uniformity in the treatment protocols because of the absence of guidelines in the literature, the heterogeneity of the cases and the involvement of multiple clinicians. Additional prospective studies with a higher number of cases comparing different treatment protocols would be interesting to evaluate their effects.

Athletic prognosis of vertebral spondylosis has been previously described as ‘guarded’ (Van Wessum 2020). Interestingly, in the present study, seven of the 11 horses for which this data was available had poor outcomes after diagnosis. All four lame horses (cases 4, 8, 9 and 10) had a poor outcome, regardless of lameness grade. Of the three cases whose outcome was described as ‘excellent’, two had grade 5 lesions. A similar trend has been reported in the literature, where more advanced lesions are generally associated with a lower level of pain (Jeffcott 1980). Two out of three of these horses had concurrent causes of pain whose resolution might have contributed to the outcome. Most of the horses with a poor outcome (six out of seven horses) presented only one lesion. Although there was not a statistically significant difference between the TS lesions grade and number of outcome categories, it is important to consider that the groups in the current study are very small and formal analysis is likely to result in a type II error. For the same reason, the outcomes were grouped in just two categories. Further studies with larger groups could potentially refute these findings; hence the results of this study need to be interpreted carefully, considering such limitations.

The main limitations of the present study consisted of the small number of cases and the lack of a common treatment protocol, as well as the presence of concurrent orthopaedic pathologies that might also be causing back pain. The population included was limited to referral cases and might therefore not represent the general population.

## 5 Conclusions

The present study reports the clinical experience in a small number of horses affected by TS. The horses more commonly affected were adult mares taking part in various disciplines, mainly jumping. Most horses were found to be affected by a single lesion, which often presented higher grade. Follow‐up radiographic assessment identified lower‐grade lesions as the faster‐evolving ones, and this was consistent with scintigraphic findings. Long‐term outcome was mostly poor. Further studies with a higher number of horses would be required to characterise this pathology further.

## Author Contributions

Claudia de Secondi and Federica Cantatore contributed to the collection of data, data analysis and writing the manuscript. Marco Marcatili, Jonathan Withers, Davide Zani and Donatella de Zani contributed to study design and contribute to data analysis. Davide Zani, Donatella de Zani and Marianna Biggi contributed to data collection and review of the images.

## Ethics Statement

Retrospective clinical study, no ethical approval required.

## Conflicts of Interest

The authors declare no conflict of interest.

### Peer Review

The peer review history for this article is available at https://publons.com/publon/10.1002/vms3.70196

## Supporting information



Supporting Information

Supporting Information

## Data Availability

Data are available on request due to privacy/ethical restriction.
