# Supplementary material for: Spondylosis in Horses: Clinical Features, Diagnostic Imaging Findings, Treatment and Outcome in 13 Horses
Source: Vet Med Sci. 2025 Mar 20;11(2):e70196. doi: 10.1002/vms3.70196 (PMC11923389; doi:10.1002/vms3.70196)
Supplement: Supplementary file 1 — Supporting Information [file VMS3-11-e70196-s001.docx]

-Which was the use of the horse before diagnosis? At which level?

-Did the horse go back to the same level of exercise after TS diagnosis?

-If yes, was the horse able to perform to higher or same level of exercise? How long after?

-If not, was the horse able to perform to lower level of exercise? Was the horse able to be ridden again or it was retired? How long after?
