# Supplementary material for: Spondylosis in Horses: Clinical Features, Diagnostic Imaging Findings, Treatment and Outcome in 13 Horses
Source: Vet Med Sci. 2025 Mar 20;11(2):e70196. doi: 10.1002/vms3.70196 (PMC11923389; doi:10.1002/vms3.70196)
Supplement: Supplementary file 2 — Supporting Information [file VMS3-11-e70196-s002.docx]

| **case** | **breed** | **age** | **sex** | **use** | **presenting complaint** | **lameness (AAEP) and diagnostic analgesia** | **TS sites** | **grade of spondylosis lesion on admission** | **concurrent orthopaedic pathologies** | **treatment** | **outcome** |
| --- | --- | --- | --- | --- | --- | --- | --- | --- | --- | --- | --- |
| **1** | ISH | 11 | M | general riding | Horse disunited in canter | NA | T11-T12 | 4 | LF enthesopathy ALDDFT + foot imbalance,  Four sites of DSPs pathology (T13-T17, one grade 1, three grade 2) | 4 months rest, 4 months groundwork, physiotherapy | excellent |
| **2** | Welsh Cob x show hunter | 14 | M | dressage | Reluctant to canter on right rein | NA | T13-T16 | 2, 4, 4 | Five sites of DSPs pathology (T14-T18, two grade 1, three grade 2) | tiludronate | excellent |
| **3** | TB X | 12 | M | eventing | Recently not moving well | NA | T12-T13 | 1 | NA | NSAIDs, 4 months hand walking, mesotherapy | good |
| **4** | ISH | 7 | G | eventing | Unwilling to go forward and stopping while hacking. | RH 1/5, no diagnostic analgesia | T13-T14 | 2 | mild osteophyte right SI, loss of homogeneity of lumbosacral disc, one site of DSPs pathology (T16-T17, one grade 1) | Tiludronate, NSAIDs (phenylbutazone, 1 gr/day for 1 month), 2 months hand walking, physiotherapy | poor |
| **5** | TB | 11 | G | general riding | Reluctance to canter, holding head up once ridden | NA | T13-T14 | 2 | Five sites of DSPs pathology (T13-T18, one grade 1, three grade 2, one grade 3) | 4 months rest, NSAIDs (phenylbutazone 2 gr/day, corticosteroid injection in 4 facet joints, mesotherapy | poor |
| **6** | WB | 13 | G | show jumping | Bucking when ridden, girthy | NA | T13-T14 | 4 | Two sites of DSPs pathology (T17-L1, one grade 2 and one grade 3). | Tiludronate, 3 months rest, then ridden flatwork physiotherapy (water treadmill) | poor |
| **7** | Dutch WB | 10 | M | eventing, hunting | NA | NA | T11-T15 | 2, 4, 5, 5 | One site of DSPs pathology (T16-T17, grade 1) | NA | NA |
| **8** | Sport Horse | 12 | M | show jumping | Reluctance to jump, toe dragging | LH 2/5, positive to deep branch of lateral plantar nerve block | T13-T16 | 2, 2, 4 | Eight sites of DSPs pathology (T11-L2, two grade 1, four grade 2, one grade 3 and two grade 4 sites) enthesopathy supraspinous ligament, hindlimbs proximal suspensory ligament desmitis. Osteoarthritis of T15 &T16 left articular facet. | Tiludronate, extracorporeal shockwave therapy, hand walking | poor |
| **9** | TB X | 14 | M | general riding | Rearing when tacked up and ridden. | RF 3/5, positive to lateral palmar nerve block | T12-T13 | 3 | DIPJ osteoarthritis | 1xtiludronate, NSAIDs (suxibuzone, 1.5 gr/ day for 2 weeks), physiotherapy | poor |
| **10** | Unknown | 17 | M | general riding | NA | LF 2/5, positive to abaxial sesamoid nerve block | T11-T12 | 2 | Five sites of DSPs pathology (T13-T18, five grade 2 sites) articular facet joints osteoarthritis C3-C7 | 1xtiludronate, NSAIDs (phenylbutazone 1 gr/ day for 2 weeks, physiotherapy | poor |
| **11** | TB | 8 | G | eventing | Back pain | NA | T11-T12 | 4 | Eight sites of DSPs pathology (T13-L2+L3-L4, two grade 1, two grade 2, two grade 3 and two grade 4 sites) | NSAIDs (phenylbutazone 1 gr/day for 4 weeks,  physiotherapy | poor |
| **12** | WB | 12 | G | Show jumping | Disunited in canter before jumps | NA | T9-T14 | 1, 5, 5, 5, 5 | Forelimbs suspensory ligament desmopathy | Tiludronate, mesotherapy | excellent |
| **13** | WB | 12 | M | Show jumping | NA | NA | T10-T16 | 1, 2, 5, 5, 5, 5 | NA | NA | NA |

Table 1B
